# Exploring memory function in earthquake trauma survivors with resting-state fMRI and machine learning

**DOI:** 10.1186/s12888-020-2452-5

**Published:** 2020-02-03

**Authors:** Yuchen Li, Hongru Zhu, Zhengjia Ren, Su Lui, Minlan Yuan, Qiyong Gong, Cui Yuan, Meng Gao, Changjian Qiu, Wei Zhang

**Affiliations:** 1 Mental Health Center, West China Hospital of Sichuan University, Chengdu, China; 2 Mental Health Center and Psychiatric Laboratory, the State Key Laboratory of Biotherapy, West China Hospital of Sichuan University, Chengdu, China; 3 Huaxi Brain Research Center, West China Hospital of Sichuan University, Chengdu, China; 4 Department of Clinical Psychology, Southwest Hospital, Army Medical University (The Third Military Medical University), Chongqing, China; 5 Huaxi MR Research Center (HMRRC), Department of Radiology, State Key Laboratory of Biotherapy, West China Hospital of Sichuan University, Chengdu, Sichuan People’s Republic of China

**Keywords:** Trauma survivor, Memory, Machine learning, fMRI, Association

## Abstract

**Background:**

Traumatized earthquake survivors may develop poor memory function. Resting-state functional magnetic resonance imaging (rs-fMRI) and machine learning techniques may one day aid the clinical assessment of individual psychiatric patients. This study aims to use machine learning with Rs-fMRI from the perspectives of neurophysiology and neuroimaging to explore the association between it and the individual memory function of trauma survivors.

**Methods:**

Rs-fMRI data was acquired for eighty-nine survivors (male (33%), average age (SD):45.18(6.31) years) of Wenchuan earthquakes in 2008 each of whom was screened by experienced psychiatrists based on the clinician-administered post-traumatic stress disorder (PTSD) scale (CAPS), and their memory function scores were determined by the Wechsler Memory Scale-IV (WMS-IV). We explored which memory function scores were significantly associated with CAPS scores. Using simple multiple kernel learning (MKL), Rs-fMRI was used to predict the memory function scores that were associated with CAPS scores. A support vector machine (SVM) was also used to make classifications in trauma survivors with or without PTSD.

**Results:**

Spatial addition (SA), which is defined by spatial working memory function, was negatively correlated with the total CAPS score (r = − 0.22, *P* = 0.04). The use of simple MKL allowed quantitative association of SA scores with statistically significant accuracy (correlation = 0.28, *P* = 0.03; mean squared error = 8.36; P = 0.04). The left middle frontal gyrus and the left precuneus contributed the largest proportion to the simple MKL association frame. The SVM could not make a quantitative classification of diagnosis with statistically significant accuracy.

**Limitations:**

The use of the cross-sectional study design after exposure to an earthquake and the leave-one-out cross-validation (LOOCV) increases the risk of overfitting.

**Conclusion:**

Spontaneous brain activity of the left middle frontal gyrus and the left precuneus acquired by rs-fMRI may be a brain mechanism of visual working memory that is related to PTSD symptoms. Machine learning may be a useful tool in the identification of brain mechanisms of memory impairment in trauma survivors.

## Background

Trauma survivors are at high risk of developing mental disorders, including a series of psychiatric symptoms (e.g., re-experiencing [1], depression [1, 2], and anxiety [1, 3, 4]) as well as cognitive decline (e.g., memory impairment [5, 6]). Nevertheless, these symptoms are difficult to measure, especially cognitive decline, which may lead to loss of well-being in later life [7].

Trauma can change brain function and structure, which may, in turn, lead to memory impairment. For example, memory impairment in mice may persist for extended periods beyond the presentation of predatory stress [8]. Besides, some studies have suggested that patients with PTSD develop impairments in working memory, delayed memory, instantaneous memory, attention, figural memory and visuospatial ability [9–11]. Furthermore, trauma survivors who express higher levels of early PTSD symptoms have impaired immediate figural memory and delayed figural memory, which indicates that they are more likely to develop full-blown PTSD in the future [12]. Previous memory models indicate that dissociation and inadequate memory encoding and processing may play a causal role in the development of PTSD [13–17]. Trauma changes the behavior and underlying neurobiology of the individual regardless of whether he or she develops PTSD [18]. Research has demonstrated that trauma survivors have more substance- and alcohol-abuse problems [19–22]. Regarding changes in underlying neurobiology, previous studies have indicated trauma-induced changes in brain function, i.e., abnormalities in the function and structure of the brain, which can lead to specific cognitive abnormalities, including memory damage and emotional processing abnormalities. For example, Lui et al. demonstrated that in participants who have survived earthquakes, the activity of the frontolimbic and striatal areas increases, while the connectivity among limbic and striatal networks decreases [23]. Earthquake survivors have been found to have a decreased gray matter volume in the bilateral insula, hippocampus, left caudate and putamen and increased gray matter volume in the bilateral orbitofrontal cortex and the parietal lobes 13–25 days after the earthquake [24]. Besides, Golub et al. found that due to the shrinkage of axonal protrusions, a traumatic experience in mice causes a reduction in hippocampal and central amygdala volumes, which are important areas in memory function [25]. Therefore, dividing trauma survivors into two groups (with and without PTSD) based on diagnostic categories can cause brain, cognition, and behavioral changes in trauma survivors without critical PTSD to go unnoticed.

To provide effective precision therapeutics for trauma survivors, it may be helpful to study the effects of trauma on the brain and on an individual’s behavior to further investigate the neuromechanistic and behavioral indicators regardless of the diagnosis, also known as the research domain criteria (RDoC) [26]. To make a contribution to the RDoC in PTSD, Gong et al. quantitatively predicted individual clinical scores of PTSD in earthquake trauma survivors using the resting-state mean amplitude of spontaneous low-frequency fluctuation (mALFF) combined with multivariate machine learning techniques [27]. The spontaneous low-frequency fluctuation (ALFF) is a neuroimaging method used to measure regional spontaneous fluctuations in the BOLD-fMRI signal intensity in fMRI, and it is the averaged square root of activity in spontaneous low-frequency (0.01–0.08 Hz) fluctuations [28]. The mALFF value reflects the degree of its raw ALFF value divided by the average ALFF value of the whole brain, which can avoid the influence of individual differences on brain activity levels [29]. Their study suggested that the prefrontal, parietal and occipital regions make a significant contribution to the association of 17-item PTSD checklist (PCL-17) scores, which challenges the traditional hypothesis that the frontolimbic network is the most important contributor to PTSD symptoms. Gong’s study was the first to suggest that RDoC can be used at the individual level and to explore the essential neuroimaging mechanism of trauma in the brain. They found that a number of regions outside this frontolimbic network, which is traditionally associated with PTSD, contribute to the association with clinical PTSD scores. However, this study only predicted PTSD symptoms.

Noticeably, memory deficits can be objective behavioral indicators related to the severity of trauma-related disorders. Elzinga et al. have indicated that both psychological and neurobiological data create a model for a trauma-related disorder as a disorder of memory [22, 30]. According to general memory models [3, 16, 30–32], inadequate memory encoding and processing can affect visual sensory information in the visual-spatial template caused by trauma-related stimuli and result in flashbacks [33] and other symptoms [34]. Therefore, it would be more efficient to explore the neuroimaging mechanisms, such as mALFF, of specific abnormal cognitive functions than to explore the relationship between symptoms and neuroimaging mechanisms. In addition, cognitive function is more measurable than symptoms estimated by a physician’s evaluation, and it could be the internal neural cognitive basis of post-traumatic mental symptoms [34], which could be used as supplementary objective criteria for PTSD clinical diagnosis in the future. Although the neuromechanisms underlying trauma-related memory defects remain unclear, it is necessary to further examine whether neuroimaging could be applied to investigate memory functions in trauma-exposed individuals.

The use of multivariate machine learning techniques [35] has been a recent way to try to increase the translational applicability of functional neuroimaging. Compared with standard mass-univariate analytical methods correlated with cognitive performance measures, these techniques can provide results at the individual level for cognitive function without clinical cognitive function measures as well as an ideal framework for investigating cognitive impairment, which could include a distributed network of regions. Importantly, we used simple multiple kernel learning (simple MKL) [36] in the current study, which is one of the MKL methods. MKL is a set of machine learning methods using a predefined set of kernels. Kernels are specified by researchers. In the current study, we defined regions of interest (ROIs) as kernels. The ROIs were defined by the AAL template (Additional file 1: Part-1). Kernel methods in MKL are class of algorithms for pattern analysis which can be operated in a high-dimensional, and learn a combination of kernels as part of the algorithm [37–39]. Simple MKL is a method that can use support vector machines (SVMs), which is supervised learning models with associated learning algorithms that analyze data used for classification and regression analysis, to simultaneously learn kernels and the associated predictor in supervised learning settings and is based on mixed-norm regularization [36].

In the present study, we used the Wechsler Memory Scale-IV (WMS-IV**)** [5] to investigate the memory function correlated with CAPS-IV [40] scores in trauma survivors. Consequently, we evaluated the potential of rs-fMRI in making accurate associations about memory function [41–46]. Then, we tried to predict the memory function related to PTSD symptoms at an individual level and to explore the mechanism underlying memory deficits. In addition, to compare the accuracy of rs-fMRI in classifying by PTSD diagnosis with the accuracy of rs-fMRI in associating PTSD symptoms with memory deficits, we applied SVM and simple multiple kernel learning (MKL) (Additional file 1: Part-2). We hypothesized that memory function, especially visual memory and working memory, is related to the CAPS-IV score. Besides, we calculated the quantitative associations of individual visual memory functions in trauma survivors by machine learning using rs-fMRI mALFF. Furthermore, we tried to explore the spontaneous activity of brain regions that could be part of the fundamental neuromechanism underlying memory deficits in trauma survivors.

## Methods

### Subjects

The study was approved by the Medical Research Ethics Committee of West China Hospital, Sichuan University, and written informed consent was obtained from all participants before the study. We acquired whole-brain resting-state fMRI for 89 individuals with a history of trauma (aged between 21 and 60 years, with an average of 45.18 years, all right-handed). According to the RDoC approach, we included subclinical PTSD to investigate the whole-brain mALFF as well as memory functions regardless of the diagnosis. The current study include (a) Individuals with CAPS-IV scores from 0 to 19 (asymptomatic/few symptoms (*n* = 41, mean = 5.60, SD = 5.97), (b) Individuals with CAPS-IV scores from 20 to 39 (subthreshold PTSD, *n* = 8, mean = 27.57, SD = 5.19), (c) Individuals with CAPS-IV scores above 39 (moderate to extreme PTSD, *n* = 40, mean = 85.33, SD =27.20) [47] (Additional file 1: Part-3). All participants were recruited seven years after the Wenchuan earthquake hit Sichuan, China; participants were recruited through the Mental Health Center of the Western China Hospital, Chengdu, China (Table 1). In addition, all participants had similar socioeconomic and cultural backgrounds. They were all residents of Qingchuan Village (the epicenter of the earthquake), and they were all present at Qingchuan Village when the earthquake occurred. In addition, all participants were interviewed to confirm the following: no history of psychiatric illness among their first-degree relatives; no history of head injury or loss of consciousness (> 1 h) or neurologic disorders; no present or past Axis-I psychiatric disorders other than PTSD; no history of psychotherapy; no learning or developmental disorders; and no history of drug or alcohol abuse in the six months preceding the scan. All subjects were assessed by DSM-IV structured clinical interview (SCID) [48] and CAPS-IV [40] by a consensus between two attending psychiatrists and a trained interviewer.

**Table 1:** Trauma survivors’ demographic data (S.D.)

	Trauma survivors
Male/female	29/60
Age (years)	45.18 (6.31)
Years of schooling (years)	8.65 (3.25)
CAPS	38.93 (37.00)

### Neuropsychological assessment

The WMS-IV [49] is a battery of tests designed to evaluate immediate and delayed recall, working memory, learning, and recognition of information that is presented in visual or verbal modalities. WMS-IV consists of five subtests: logical memory subtest (LM) to assess the narrative memory, vocabulary paired association (VPA) to evaluate speech memory, design (DE) to assess the sensory visual stimulation, visual reproduction (VR) to measure the memory of nonverbal visual stimuli, and spatial addition (SA) to evaluate visual working memory. The primary subtest yields four WMS-IV index scores (visual working memory, auditory memory, visual memory, and delayed memory) and an overall full-scale memory quotient (Additional file 1: Part-4).

### *Image Acquisitio*n

Experiments were performed on a 3.0 T magnetic resonance scanner (Siemens 3.0 T Trio Tim, Germany) with an eight-channel phased-array head coil. Functional images were acquired using a single shot, gradient-recalled echo-planar imaging sequence (repetition time (TR)/echo time (TE) = 2000/30 ms; flip angle = 90°). Five dummy scans were collected prior to the actual MRI scans, and the first 5 volumes of the MRI time series were discarded for magnetization stabilization. The slice thickness was 5 mm with no gap, 64 × 64 matrix size and a field of view of 240 × 240 mm^2^, resulting in a voxel size of 3.75 × 3.75 × 5 mm^3^. Each brain volume comprised 30 axial slices, and each functional run contained 205 image volumes. Subjects were instructed to relax, keep their eyes closed, and let their minds wander without falling asleep during the 6.8 min scan [50].

### Data preprocessing

The data were preprocessed using Statistical Parametric Mapping (SPM8, http://www.fil.ion.ucl.ac.uk/spm). The first 5 volumes were discarded for scanner calibration and participant acclimation to the scanning conditions. All data were corrected for slice timing. Besides, head motion correction of the functional scans was performed for the remaining 200 consecutive volumes. The three translational and three rotational motion parameters were first computed during the realignment step. Then, we generated the mean framewise displacement (FD), which reflected the volume-to-volume changes in the head position [51]. Data from 9 trauma survivors in 98 subjects were discarded when the mean FD exceeded 0.25 mm or when translational or rotational parameters exceeded ±1.5 mm or ± 1.5°. Therefore, 89 subjects were included in the analysis of the current study. Demographic data and clinical symptom scores of 98 subjects were shown in Additional file 1: Part-5. We coregistered high-resolution structural images to the mean functional image and segmented them into white matter, gray matter and cerebrospinal fluid in MNI space using “new-segment and DARTEL” in the data processing assistant for resting-state fMRI (DPARSF) after slice timing and realignment. Nuisance covariates, including the head motion (Friston 24-parameter model) [52], cerebrospinal fluid signals and white matter signals, were regressed out. Next, we removed the linear trend after spatial normalization. Finally, the images were normalized to the MNI space (voxel size: 3 mm^3^) with the DARTEL tool, and images were smoothed using a 6-mm full width half maximum (FWHM) isotropic Gaussian filter.

### ALFF calculation

#### Temporal filtering

The result data were further temporally bandpass filtered (0.01–0.08 Hz) to reduce the effects of low-frequency drift and high-frequency physiological noise. The time series were transformed into the frequency domain using the Fourier transform (FFT) to obtain the power spectrum after linear-trend removal. To calculate the ALFF, the square-rooted power spectrum was obtained. Then, the ALFF of each voxel was divided by the global mean of ALFF values for standardization to obtain the mALFF, which was calculated using the data processing and analysis for brain imaging (DPABI) [53] (http://rfmri.org/dpabi) software.

### Statistical measures

The score and subscale scores of the CAPS_IV and cognitive test scores were analyzed by partial correlation analyses, controlling for the potential influences of age, gender, and educational background, achieving multiple comparison corrections by Bootstrap analysis. *P* < 0.05 was considered to be a statistically significant difference. All tests were performed using SPSS software, version 17.0 for Windows.

### Univariate SPM analysis

A standard, univariate approach was used in SPM8 software to investigate the relationship between the resting-state mALFF and the memory subscale scores that are significantly correlated with CAPS-IV scores.

### Simple multiple kernel learning analysis

The relationship between the cognitive test scores and the mALFF at rest were examined using simple MKL [36] as implemented in PRoNTo v2.1 (http://www.mlnl.cs.ucl.ac.uk/pronto/) running under MATLAB (Mathworks, 2010 release). The simple MKL algorithm is based on the objective value of gradient descent on the support vector machine (SVM). Then, gradient descent wrapping is used to iteratively determine the combination of kernels [36]. In the current study, a linear combination of 116 basic kernels was considered for the final synthetic nuclear space. We also regress out the mean FD in the analysis, a leave-one-out cross-validation was applied across participants to obtain estimates for each participant. The accuracy of simple MKL association was calculated, defined as the Pearson’s correlation coefficient and the mean squared error (MSE) between the actual and predicted values of the cognitive scores (Additional file 1: Part-6).

A randomization test, defined as a permutation test, was used to estimate the distribution of correlation and MSE values under the null hypothesis of no association between mALFF and cognitive ability by randomly pairing the input-target data and the simple MKL rerun 1000 times, which provided an estimated *P*-value for both the correlation coefficient and the observed MSE.

To facilitate visualization, the current study established a table to sort the contribution of different ROIs to the size of the synthesis of the nuclear space. The expected ROI of different ROIs sorted by leave-one-out cross-validation is calculated by the synthetic kernel space. The smaller the expected sort value, the larger the average weight of the ROI is and the higher the ranking is.

### Support vector machine (SVM) classification analysis

The mALFF classification of trauma survivors with (CAPS_IV score ≥ 40) or without PTSD (CAPS_IV score < 20) was examined using simple SVM [54] as implemented in PRoNTo running under MATLAB. SVM was used to investigate the potential of whole-brain mALFF for discriminating among trauma survivors with or without PTSD. Besides, the multi-kernel combination strategy [55] was used to effectively combine different feature vectors. The weights of different kernels in the SVM were learned based on the training samples [36]. We also regress out the mean FD in the analysis.

A leave-one-out cross-validation was applied to validate the performance of our proposed approach. Thus, when each sample was designated as a test sample (all other samples were left out for the test), the remaining samples were used to train the classification function. In this manner, it could derive an approximately unbiased estimator of the model. To quantify the performance of compared methods, balanced accuracy, sensitivity and specificity are reported, which are defined based on the association results of LOOCV. Balanced accuracy takes the number of samples in each class into account, and it gives equal weight to the accuracies obtained on the test samples of each class.

In the discrimination maps, each voxel carried a certain weightage, indicating its contribution to the classification function. In this way, a discrimination map could be generated.

The details on the kernels and the information on the tuning parameters are in the Additional file 1: Part 1.

## Results

### Psychological and behavioral data

The study included 89 subjects. Subject demographic data and clinical symptom scores are shown in Table 1.

### Correlation between cognitive dimensions and CAPS_IV scale

Table 2 shows the correlations between cognitive performance and the symptoms assessed by CAPS_IV for the trauma survivors. Most of the cognitive functions evaluated in the WMS-IV did not correlate with the total scores of CAPS_IV. The spatial addition (SA) subtest is the only subtest that displayed a negative correlation with the total CAPS_IV scores. The visual memory index, visual working memory index, immediate memory index, visual reproduction, and SA displayed a negative correlation with the symptoms of re-experiencing as measured by CAPS_IV. Moreover, SA displayed a negative correlation with the symptoms of avoidance as measured by CAPS_IV.

**Table 2:** The correlation between CAPS score and cognitive function by bootstrap

Value	CAPS	
	Correlation	*p*
LM (instant)	−0.18	0.10
LM (delayed)	0.02	0.86
VPA (instant)	−0.04	0.69
VPA (delayed)	−0.03	0.79
VPA Recognition (delayed)	0.06	0.60
VR (instant)^b^	−0.23	0.12
VR (delayed)	−0.15	0.24
DE (instant)	−0.21	0.11
DE (instant) Content	−0.21	0.06
DE (instant) Space	−0.17	0.13
DE (delayed)	−0.17	0.12
DE (delayed) Content	−0.11	0.32
DE (delayed) Space	−0.08	0.47
SA	−0.22	0.04*

Abbreviation: *CAPS* Clinician-Administered Posttraumatic Stress Disorder Scale, *LM* Logical Memory, *VPA* Vocabulary paired association, *VR* Visual reproduction, *DE* Design, *SA* Spatial Addition

*significant by bootstrap analysis (*p* < 0.05)

### Univariate SPM analysis

There are no regions that showed a significant association with SA scores at *P* < 0.05 (corrected for multiple comparisons using family-wise error (FWE)) (Additional file 1: Part 7).

### Simple MKL analysis

**Fig. 1:**
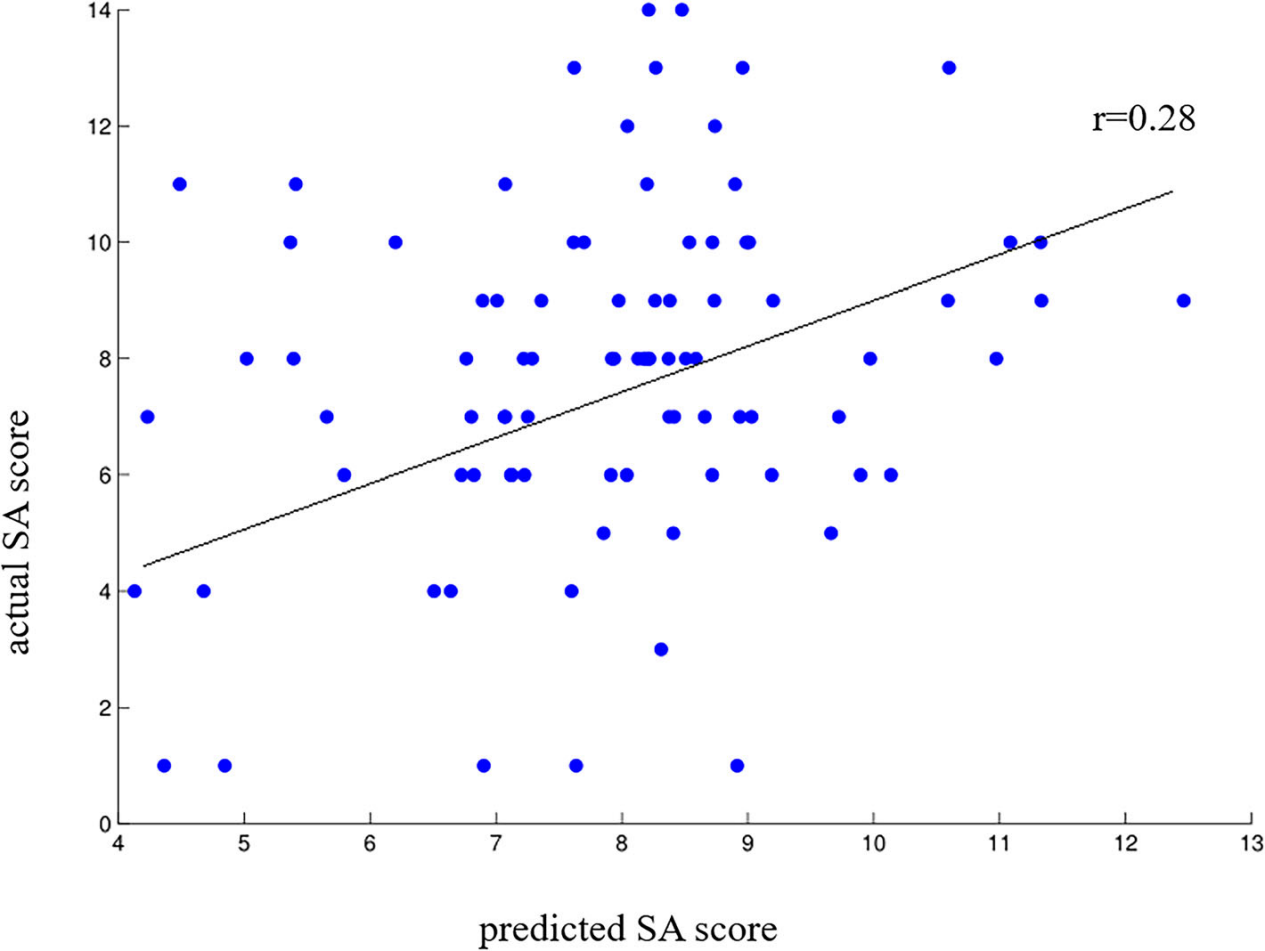
Scatter plot showing the predicted SA score for each subject derived from their resting-state mALFF data using simple MKL, vs. their actual SA score, SA, Spatial Addition; mALFF, mean amplitude of spontaneous low Frequency; MKL, Multiple kernel learning analysis

**Fig. 2:**
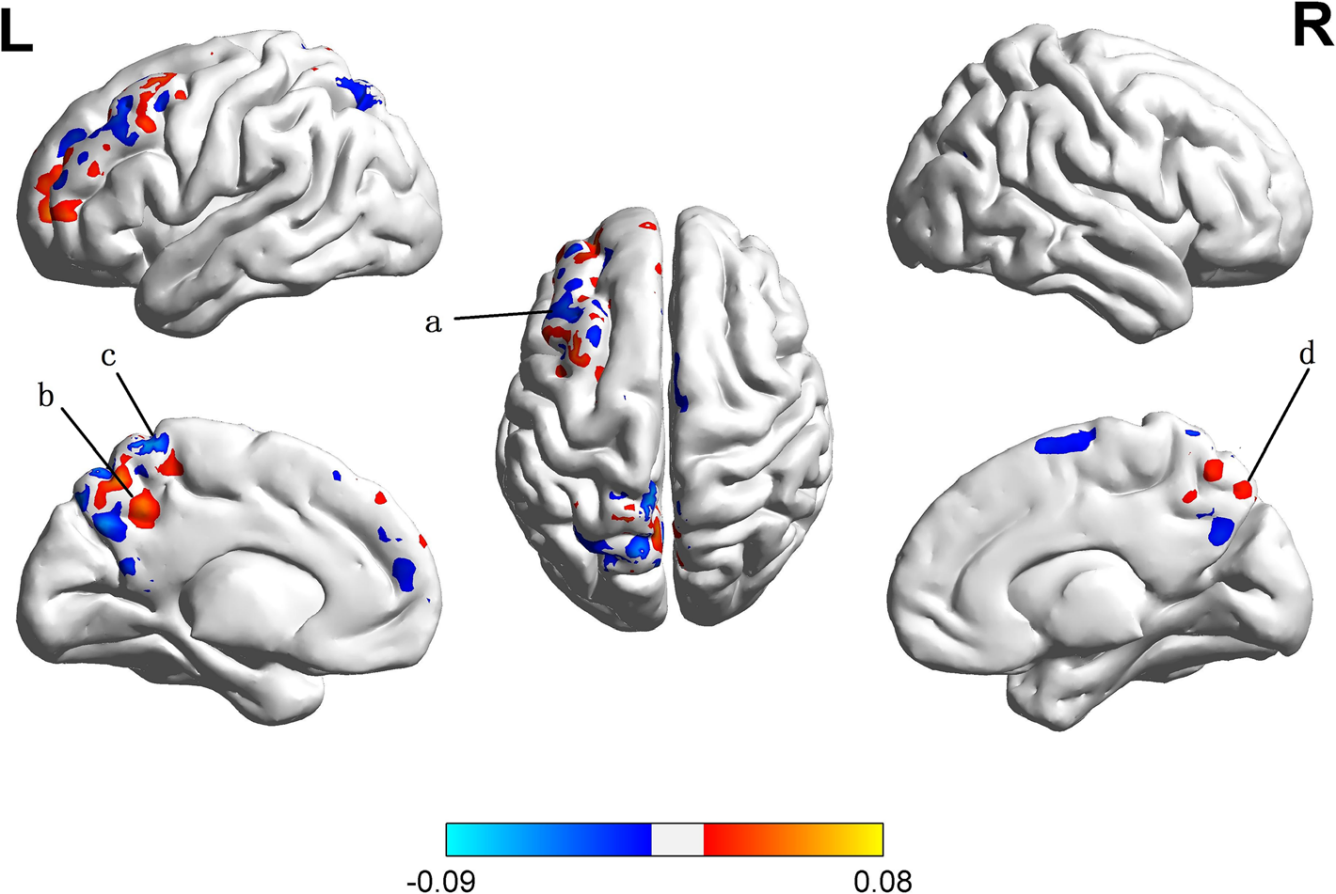
Multivariate map showing the weight of each part of brain region indicating its relative contribution to the regression function in the context of all other brain regions (color bar in arbitrary units). [**a**] left frontal middle gyrus; [**b**] left precuneus;[**c**] left superior parietal gyrus;[**d**] right precuneus

As mentioned above, the SA subtest is the only subtest that displayed a negative correlation with the total CAPS_IV scores. The application of simple MKL allowed quantitative association of SA scores by whole-brain resting-state mALFF in statistically significant accuracy (correlation = 0.28, *P*-value = 0.026; mean squared error = 8.36, P-value = 0.035) (corrected for multiple comparisons using the permutation test, both *P* < 0.05 was the significant level) (Fig. 1) in trauma survivors. The association was based on functional alterations across the whole brain, particularly in the left frontal middle gyrus and left precuneus, in addition to parietal lobes and occipital regions (Table 3**,** Fig. 2, and Additional file 1: Part 8). Table 3 expresses the neuroanatomical regions with a contribution to the association frame above 2% across all regions for the rs-fMRI-based MKL used to accurately predict SA. However, the simple MKL to the whole-brain resting-state mALFF data failed to make a statistically significant accurate quantitative association of SA scores (correlation = − 0.04, *P*-value = 0.497; mean squared error = 9.25, *P*-value = 0.712) (corrected for multiple comparisons using a permutation test, both *P* < 0.05 was the significant level) in PTSD patients (CAPS_IV score ≥ 40).

**Table 3:** Weighted sorting and expected sorting table

AAL	Brain region	Contribution proportion (%)	Number of voxels (vox)	Desired ordering
7	Frontal_Mid_L	23.895726	1388	1.022472
67	Precuneus_L	20.261179	945	1.94382
59	Parietal_Sup_L	8.929654	546	3.213483
68	Precuneus_R	8.092137	898	4.067416
8	Frontal_Mid_R	7.138229	1159	5.123596
23	Frontal_Sup_Medial_L	6.70388	833	5.797753
20	Supp_Motor_Area_R	5.655513	668	6.764045
52	Occipital_Mid_R	3.450836	565	8.41573
3	Frontal_Sup_L	2.784355	1013	9.977528
14	Frontal_Inf_Tri_R	2.386954	463	10.202247
4	Frontal_Sup_R	2.069247	993	11.696629
85	Temporal_Mid_L	2.045613	1421	15.561798

Abbreviation: Frontal_Mid_L,Left frontal middle gyrus; Precuneus_L,Left precuneus; Parietal_Sup_L,Left superior parietal gyrus; Precuneus_R,Right precuneus; Frontal_Mid_R,Right middle frontal gyrus; Frontal_Sup_Medial_L,Left superior frontal gyrus,medial; Supp_Motor_Area_R,Right supplementary motor area; Occipital_Mid_R,Right Middle occipital gyrus; Frontal_Sup_L,Left superior frontal gyrus,dorsolareral; Frontal_Inf_Tri_R,Right inferior frontal gyrus,triangular; Frontal_Sup_R,Right superior frontal gyrus,dorsolateral; Temporal_Mid_L,Left Middle temporal gyrus;

### SVM analysis

In discriminating among trauma survivors with moderate to extreme PTSD (*n* = 40) or with asymptomatic/few symptoms (*n* = 41), the SVM analysis of the whole-brain resting-state mALFF data failed to classify trauma survivors with a statistically significant accuracy (Total accuracy = 55.56%, balanced accuracy = 55.46%, *P* value = 0.142; sensitivity =47.50%, *P* value = 0.42; specificity =63.41%, *P* value = 0.103; positive predictive value = 55.88%; negative predictive value = 55.32%; kappa = 0.158) (corrected for multiple comparisons using permutation test, both *P* < 0.05 was the significant level).

## Discussion

The current study indicated that the SA score is the only subtest score in WSM-IV that is associated with PTSD symptoms. The current study explored the quantitative association of visual working memory by whole-brain resting-state mALFF at the individual level with statistically significant accuracy. It examined the pathophysiological mechanisms of the visual working memory in trauma survivors. Importantly, the current work used a model that predicts visual working memory and further studied the neuromechanisms of visual working memory in the distributed network of brain regions of trauma survivors, laying the foundation for RDoC [56].

This study found that visual working memory was negatively correlated with the severity of PTSD symptoms (see Additional file 1: Part 9). These results are consistent with previous studies [57]. Stein et al. suggested that visual-spatial ability is significantly reduced in patients with PTSD who experienced rape compared with that in healthy controls [57]. Besides, among war asylum seekers, the visual-spatial ability in patients with PTSD is significantly lower than that in war survivors without PTSD [58]. Moreover, a negative relationship has also been found between PTSD symptom severity and working memory performance after controlling for age, gender and educational background [56]. We can speculate that the visual working memory impairment may have a detrimental role in the failure of visual information processing [3, 16, 30–32] and that the visual working memory impairment is responsible for disrupted declarative memory of visual information [59, 60], leading to PTSD symptoms [61]. Consequently, our data indicated an important role for visual working memory in PTSD.

In the present study, we extended these early results to show that the application of simple MKL in the rs-fMRI can be used for the quantitative association of cognitive scores by whole-brain resting-state mALFF at the individual level in statistically significant accuracy. In recent work on trauma survivors, the combination of multivariate machine learning techniques and rs-fMRI showed the potential for predicting and investigating neuromechanisms at the individual level [27]. The failure in predicting SA scores at the individual level in PTSD patients may be due to the small sample size. Moreover, as the mean score of SA was much lower in PTSD patients, a floor effect may have also resulted in the failure to predict these scores, but the influence might not be large (see Additional file 1: Part 10). This further indicates the importance of the current study, which explored a way to measure memory by neuroimaging, which can be fast, accurate and appropriate for most people. Interestingly, the low accuracy classification of PTSD in trauma survivors is consistent with previous studies. Qiongmin Zhang et al. [62] classified trauma survivors with or without PTSD with low accuracy (the best accuracy was 64.86%) when using ALFF. Although they applied a combined multimodal feature approach (combining Reho, GMV, and ALFF), the accuracy improved to only 67.57% with a sensitivity of 52.94% [62], but this accuracy is still insufficient. In contrast, in the identification of PTSD versus healthy controls (HCs), 89.19% of individuals were correctly assigned to the appropriate diagnostic category, which is much better than the identification of trauma survivors with or without PTSD [62]. In addition, when Gong et al. [63] compared survivors with or without PTSD, they found that gray matter allowed discrimination with an accuracy of 67% (*p* < 0.001); however, the two groups could not be distinguished based on white matter. This accuracy is much lower than that obtained when comparing PTSD and HCs 76% (p < 0.001) and 85% (p < 0.001) based on gray and white matter, respectively) [63]. Moreover, the application of MKL to the whole-brain resting-state mALFF data did not allow a quantitative association of CAPS_IV scores (see Additional file 1: Part 10) in the current study. This evidence indicates that it may be better to predict or classify based on RDoC than on the diagnosis, and it is more efficient to explore the whole-brain resting-state mALFF of specific abnormal cognitive functions than to explore the relationship between symptoms and whole-brain resting-state mALFF.

The current study found that the left medial frontal gyrus and bilateral precuneus, but mainly the left precuneus (the left precuneus contributed 20% to the association frame versus the 8% of the right precuneus), both of which are in the default mode network (DMN), contributed to a substantial proportion of the association model of SA score and the whole-brain resting-state mALFF in the current study. The DMN is associated with autobiographical memory, stimulus independence and internally focused thought [64]. Specifically, the precuneus is involved in memory processing and spatial location encoding [64, 65]. Moreover, previous studies have observed that greater activation in the left medial frontal gyrus is involved in the processing of working memory [66, 67]. Besides, our results indicated that the frontal lobe and temporal lobe contribute substantially to the association frame, which is consistent with our previous study [62]. Geuze et al. found that, compared with healthy controls, during the coding phase, patients with PTSD showed underactivation of the frontal cortex and overactivation of the temporal cortex [68]. In the retrieval process, decreased activation of the right frontal cortex, the bilateral middle temporal gyri, and the left posterior hippocampus/parahippocampal gyrus in veterans with PTSD were observed [68]. This difference suggests the possible involvement of the frontal lobe - temporal lobe network in the cognitive deficits seen in PTSD [68], which is in line with the current findings.

Our previous study found that the gray matter volume (GMV) difference of the bilateral middle occipital gyrus, left superior frontal gyrus and bilateral middle frontal gyrus are the most discriminative regions for distinguishing PTSD from HC [62]. Gong et al. have suggested that PTSD and HC could be discriminated based on gray and white matter in several prefrontal, temporal, parietal and occipital regions [63]. In the current study, the functions of these regions are also important to the association frame, which indicated that these regions are vulnerable brain regions related to the dysfunction of trauma survivors, not only for GMV prediction but also for ALFF estimation. This finding indicated that it may be more sensitive in finding the trauma-related brain regions by predicting the SA score. However, previous studies have been limited to the symptoms, for example, predicting whether the individuals have PTSD or not [62].

In another study, Gong et al. employed multivariate machine learning techniques for the quantitative association of clinical scores (PCL-17) by whole-brain resting-state mALFF in trauma survivors between 10 and 15 months after the event with statistically significant accuracy (correlation = 0.32, *P*-value = 0.006; the mean sum of squares = 176.88, P-value = 0.001) [27]. The accuracy in this study was higher compared with the current work, which may be due to the smaller sample size used in the current study compared to Gong’s study (188 trauma survivors) and the current study investigated trauma survivors 7 years after the event, the long time may also plays a role in reduce the accuracy. Nevertheless, the present study aimed to investigate trauma survivors 7 years after the event; all subjects were assessed by SCID [48], CAPS_IV [40] and the neuropsychological assessment performed by psychiatrists aimed to predict long-term prognostic indicators of trauma survivors (such as CAPS_IV score and memory performance) by machine learning. Noticeably, whole-brain ALFF predicted SA more accurately than it predicted the scores of the CAPS_IV scale **(**Additional file 1: Part-11). We can speculate that the relationship between neuroimaging and cognitive function is more significant than that between neuroimaging and symptoms. Additionally, the results indicated that during a long time, the neuroimaging of ALFF was more relevant to the SA function than the PTSD symptoms in trauma survivors. Gong et al. demonstrated that ALFF functional activation in several prefrontal, parietal, and occipital regions is the basis of accurate prediction, which is in line with the current study [27]. In the present study, the left frontal middle gyrus and parietal lobes contributed above 32% to the frame. The results indicated the importance of the frontal-parietal network in predicting visual working memory. Saar-Ashkenazy et al. have reported that the brain activation patterns of visual working memory in survivors with PTSD are different from those of non-PTSD trauma survivors [69]. The frontal cortex is structurally and functionally associated with the parietal lobe [70], regulating spatial memory and visual-spatial processing [71, 72]. Furthermore, Clark et al. have suggested that the encoding of visual working memory in trauma survivors with PTSD is more dependent on the spatial coding ability of the parietal lobe and less dependent on the executive control function of the frontal lobe [73]. Therefore, the dysfunctional network between the frontal lobe and parietal lobe may be the pathophysiological mechanism behind the disorder of visual working memory and post-traumatic mental disorder, especially PTSD; this current finding is in line with previous findings. The relationship between the prefrontal and parietal lobes and whether the frontal-parietal network is a common mechanism behind PTSD visual working memory impairment and PTSD symptoms needs to be further investigated.

Noticeably, in addition to the brain regions found in the above-reported studies, the current work found that the supplementary motor area (SMA) had a certain proportion of the association of SA, especially the right SMA that had an association weight of over 5% in the association model. MacNamara et al. found that patients with PTSD increased the activation of the motor area in the process of emotional regulation, unlike trauma survivors without PTSD [74]. SMA is believed to have a key role in the network of neural regions mediating top-down control of negative affect [75], and it may be involved in implementing dorsolateral prefrontal cortex-initiated emotion regulatory effects [75]. Cunnington et al. found that SMA is essential in the early component of premovement activity, which is strongly influenced by higher cognitive factors [76]. In addition, Whalley et al. found that individuals with PTSD exhibited flashback-specific increases in the SMA [77].

In contrast, the univariate analysis of the resting-state mALFF data did not reveal any regions that were significantly associated with SA scores, which is in line with a previous study [22] that found that univariate analysis was not significantly associated with clinical scores while multivariate methods were. This may be due to possible sample heterogeneity of mALFF at the group level, for example, the heterogeneity of brain regions and the individual; however, the algorithm is applied to separate individuals, so it is not a problem in machine learning analysis [55]. In addition, the standard univariate approach explored linear correlations; however, some correlations could be found in nonlinear relationships [55]. For example, in the current study, we applied nonlinear transformations by using a kernel function. The results suggest that multivariate methods are more sensitive to the stable diffuse alterations observed in psychiatric disorders. Thus, compared with standard mass-univariate techniques, multivariate methods are more suitable for development as a real-world clinical diagnostic tool [35].

Importantly, Lianne et al. found that multivariable machine learning techniques, which allow for individual associations based on high-dimensional data, are more sensitive to spatially distributed effects [78] and changes in brain regions exposed to trauma compared with standard quality - single variable techniques [54] used in previous studies [27]. Therefore, these multivariable machine learning techniques might be more suitable for clinical application. Moreover, the simple MKL showed the results related to the average contribution of each brain region to the model, rather than just showing these regions by setting the threshold to 30% of the maximum weight value [79].

## Limitations

This study has several limitations as well. One limitation is the use of cross-sectional study design with participants who were exposed to an earthquake. It is not possible to determine whether the observed cognitive function and the variability in brain function reflect potential pre-existing plastic changes in individual psychological vulnerability or if they occurred after the earthquake. Another limitation is that, because gender is one of the risk factors of PTSD, the potential neuropsychological mechanism of PTSD may differ, and future research needs to separate these mechanisms based on gender. Moreover, both the SA score and the resting-state mALFF may be influenced by the symptoms and age; however, as the covariates that are correlated with the targets could not be regressed out in the model—as this could lead to biases (positive or negative) in the obtained results [80], we could not exclude the influence of age and PTSD symptoms on the model. We are also aware that the effect size in our study is not large; however, if we exclude individuals with a very low SA score (SA = 1), the effect size of the frame is much better (correlation = 0.36, *P*-value = 0.010; mean squared error = 5.09, *P*-value = 0.010; corrected for multiple comparisons using the permutation test, both *P* < 0.05 was the significant level) **(**Additional file 1: Part-12), but we do not have the evidence to exclude them. Thus, larger sample size is needed in the future, and the influence of outliers will then be reduced. Besides, the multimodal prediction is a promising field. For example, we will explore whether both rs-fMRI and SA could inform PTSD symptoms in the future. Moreover, although calculating permutation confidence intervals was a computationally difficult problem, a method for performing this calculation in the two-sample problem was presented [81]. However, as far as we know, there is no software package in neuroimaging machine learning that can do this. Besides, we only explored the association frame based on the AAL brain atlas. No consensus atlas has been proven to be far superior to others; AAL remains the most-used atlas in machine learning analysis, although it has many drawbacks. Although AAL is widely accepted in neuroimaging studies, new brain atlases have frequently been used in neuroimaging and machine learning studies, such as the Power 264-region atlas [82, 83] and the Dosenbach’s 160 functional atlas [84]. Future studies should verify our results using these atlases in the analyses of different brain networks. Finally, although the LOOCV was helpful in developing the association model and discovering the critical features derived from rs-fMRI, it increases the risk of overfitting. However, in the current study, LOOCV was adopted to prevent the training set from deviating too much from the overall population for the relatively smaller sample (89 subjects in total) of subjects included in our study. Out-of-sample validation could be the best method to use in this study, but we did not include it in the study design. In the future, we will increase the sample size so that out-of-sample validation could be used in the study to avoid the risk of overfitting.

## Conclusion

In conclusion, this study investigated the association between memory and PTSD symptoms, and it indicated that visual working memory impairment was related to PTSD symptoms. We also explored individual associations between mALFF and visual working memory—which may be suitable for development as a real-world clinical memory assistive assessment tool—and the neuromechanism of the spatial overlay dimension of traumatic exposure using the multicore learning method. In addition, our findings indicated that, from the perspective of the whole-brain pattern, brain mechanisms (found in the frontal cortex and parietal cortex) largely contribute to the impairment of visual working memory of trauma survivors, which, to our knowledge, has not been reported before this study. These brain areas are related to memory processing and spatial location encoding. The failure to process visual memory may be due to the dysfunction of these brain areas, which in turn may be related to the symptoms of PTSD [13–17]. This indicates that RDoC studies are more effective in discovering mechanisms than studies that pay more attention to individuals with PTSD diagnosis. Working memory-related training may benefit the functioning of these brain areas [85] and may be helpful to trauma-exposed people.

## Supplementary information

**Additional file 1**. Supplementary material containing 12 parts used to prove the results of this article.

## Data Availability

The datasets used and/or analyzed during the current study are available from the corresponding author on reasonable request.

## Abbreviations

CAPS: Clinician-Administered post-traumatic stress disorder Scale
DE: Designs
DSM-5: Statistical Manual of Mental Disorders
FWE: Family Wise Error
HAMA: The Hamilton Anxiety Rating Scale
HAMD: Hamilton Depression Rating Scale
HC: Healthy control
LM: logical memory subtest
LOOCV: Leave One Out Cross-Validation
mALFF: The mean amplitude of spontaneous low-frequency fluctuation
MKL: Multiple kernel learning
PCL-17: 17-item PTSD Checklist
PTSD: Post-traumatic stress disorder
RDoC: Research Domain Criteria
ROI: Regions of interest
rs-fMRI: Resting-state functional magnetic resonance imaging
SA: Spatial Addition
SCID: DSM-IV Structured Clinical Interview
SVM: Support vector machine
VPA: Vocabulary paired association
VR: Visual reproduction
WMS-IV: Wechsler Memory Scale-IV
