# Supplementary material for: Exploring memory function in earthquake trauma survivors with resting-state fMRI and machine learning
Source: BMC Psychiatry. 2020 Feb 3;20:43. doi: 10.1186/s12888-020-2452-5 (PMC6998246; doi:10.1186/s12888-020-2452-5)
Supplement: Supplementary file 1 — Additional file 1. Supplementary material containing 12 parts used to prove the results of this article. [file 12888_2020_2452_MOESM1_ESM.docx]

**Supplementary material**

1. Details on the kernels and information on the tuning parameters

**Table S1. Regions of interest (ROIs) included in AAL-atlas**

| **Index** | **Regions** | **Abbr.** | **Index** | **Regions** | **Abbr.** |
| --- | --- | --- | --- | --- | --- |
| (1,2) | Precental gyrus | PreCG | (59,60) | Superior parietal gyrus | SPG |
| (3,4) | Superior frontal gyrus, dorsolateral | SFGdor | (61,62) | Inferior parietal, but  supramarginal and angular gyri | IPL |
| (5,6) | Superior frontal gyrus, orbital part | ORBsup | (63,64) | Supramarginal gyrus | SMG |
| (7,8) | Middle frontal gyrus | MFG | (65,66) | Angular gyrus | ANG |
| (9, 10) | Middle frontal gyrus, orbital part | ORBmid | (67,68) | Precuneus | PCUN |
| (11,12) | Inferior frontal gyrus, opercular part | IFGoperc | (69,70) | Paracentral lobule | PCL |
| (13,14) | Inferior frontal gyrus, triangular part | IFGtriang | (71,72) | Caudate nucleus | CAU |
| (15,16) | Inferior frontal gyrus, orbital part | ORBinf | (73,74) | Lenticular nucleus, putamen | PUT |
| (17,18) | Rolandic operculum | ROL | (75,76) | Lenticular nucleus, pallidum | PAL |
| (19,20) | Supplementary motor area | SMA | (77,78) | Thalamus | THA |
| (21,22) | Olfactory cortex | OLF | (79,80) | Heschl gyrus | HES |
| (23,24) | Superior frontal gyrus, medial | SFGmed | (81,82) | Superior temporal gyrus | STG |
| (25,26) | Superior frontal gyrus, medial orbital | ORBsupmed | (83,84) | Temporal pole: superior  temporal gyrus | TPOsup |
| (27,28) | Gyrus rectus | REC | (85,86) | Middle temporal gyrus | MTG |
| (29,30) | Insula | INS | (87,88) | Temporal pole: middle  temporal gyrus | TPOmid |
| (31,32) | Anterior cingulate and  paracingulate gyri | ACG | (89,90) | Inferior temporal gyrus | ITG |
| (33,34) | Median cingulate and  paracingulate gyri | DCG | (91,92) | Cerebellum_Superior | Cerebelum_Crus1 |
| (35,36) | Posterior cingulate gyrus | PCG | (93,94) | Cerebellum_Inferior | Cerebelum_Crus2 |
| (37,38) | Hippocampus | HIP | (95,96) | Cerebellum_Superior | Cerebelum_3 |
| (39,40) | Parahippocampal gyrus | PHG | (97,98) | Cerebellum_Superior | Cerebelum_4_5 |
| (41,42) | Amygdala | AMYG | (99,100) | Cerebellum_Superior | Cerebelum_6 |
| (43,44) | Calcarine fissure and  surrounding cortex | CAL | (101,102) | Cerebellum_Inferior | Cerebelum_7b |
| (45,46) | Cuneus | CUN | (103,104) | Cerebellum_Inferior | Cerebelum_8 |
| (47,48) | Lingual gyrus | LING | (105,106) | Cerebellum_Inferior | Cerebelum_9 |
| (49,50) | Superior occipital gyrus | SOG | (107,108) | Cerebellum_Inferior | Cerebelum_10 |
| (51,52) | Middle occipital gyrus | MOG | (109,110) | Vermis | Vermis_1_2, Vermis_3 |
| (53,54) | Inferior occipital gyrus | IOG | (111,112) | Vermis | Vermis_4_5, Vermis_6 |
| (55,56) | Fusiform gyrus | FFG | (113,114) | Vermis | Vermis_7, Vermis_8 |
| (57,58) | Postcentral gyrus | PoCG | (115,116) | Vermis | Vermis_9, Vermis_10 |

2. Simple MKL

Multiple Kernel Learning (MKL)^[1]^, a novel algorithm for solving the multiple kernel learning problem, which involve multiple and heterogeneous data sources. Recent applications have shown that using multiple kernels can enhance the interpretability of the decision function and improve performances than a single one ^[2, 3]^.MKL can combine different models, simultaneously learn these models, represented by different kernels, in supervised learning settings^[1]^.

3.Subjects characters


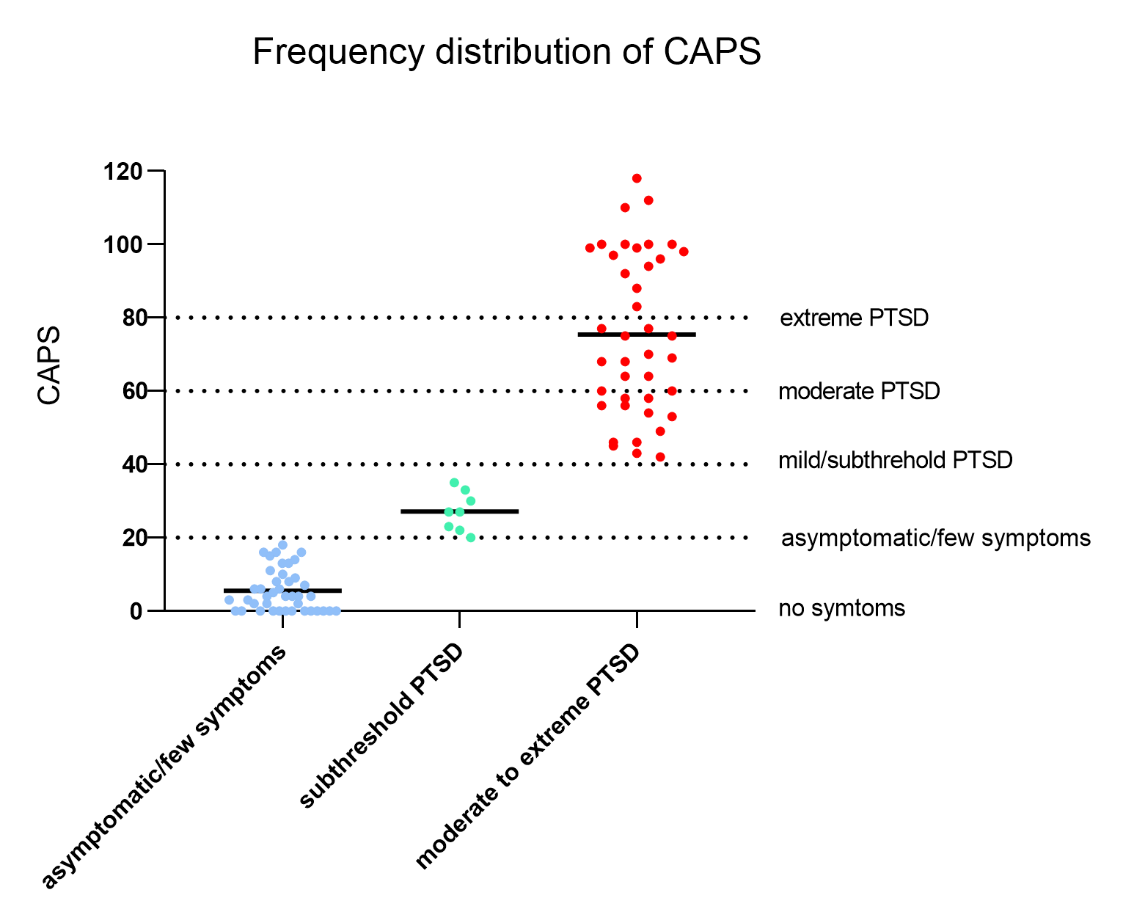


**PTSD**, post-traumatic stress disorder; **CAPS**, Clinician-Administered Posttraumatic Stress Disorder Scale

1. Memory subtest

Logical memory subtest（LM）:The subtest is to assess the narrative memory in a free conference context. The participants were required to recall for a short story. The test is divided into two parts including immediate memory and delayed memory.

Vocabulary paired association（VPA）: The subtest uses pairs of words to evaluate speech memory. The participants were required to recall for related and unrelated word pairs. The test is divided into two parts including immediate memory and delayed memory.

Designs（DE）: The subscale is used to assess the spatial significance of subjects' sensory visual stimulation. The participants were required to recall of spatial locations and visual details. The test is divided into two parts including immediate memory and delayed memory.

Visual reproduction（VR）: The subtotal is used to assess the memory of nonverbal visual stimuli. The participants were required to recall of geometric designs. The test is divided into two parts including immediate memory and delayed memory.

Spatial Addition（SA）: Use the visual superposition task to evaluate visual working memory according to this subtest. The participants were tested to know the ability to manipulate visual spatial information in working memory).

In SA subtest, first, there was one picture with several dots in the nine-box, we will tell the participants that remember the place and the number of the black dots but ignore the red ones. Second, the second picture with several dots in the nine-box present, and we will tell the participants that remember the place and the number of black dots but ignore the red ones. Participants have 5 seconds to remember each picture. Last, we will tell the participants to image overlapping the two pictures and put the dots in the grid that ever appeared in the two pictures. Besides, if the two pictures all have a dot in one grid, change the color of the dots to white. All the measures were applied in a computer by WSM-IV program.

Below is the sketch map.

Picture 1, appeared 5 seconds

|  |  |  |
| --- | --- | --- |
|  |  |  |
|  |  |  |

Picture 2, appeared 5 seconds

|  |  |  |
| --- | --- | --- |
|  |  |  |
|  |  |  |

The correct addicted map which needs participants to select and put dots in (ignore red one, overlapped black one in white):

|  |  |  |
| --- | --- | --- |
|  |  |  |
|  |  |  |

5. Demographic data and clinical symptom scores of all the subject including 9 trauma survivors were discarded

Trauma survivors’ demographic data (S.D.)

|  | **Trauma survivors** |
| --- | --- |
| male/female | 32/66 |
| Age (years) | 45.13 (6.26) |
| Years of schooling (years) | 8.69 (3.23) |
| CAPS | - 1. (37.02) |

1. The parameters and steps in the machine learning

In current study, we use the ALL template (Anatomical Automatic Labeling), which provided by the Montreal Neurological Institute (MNI) to define brain regions, which were defined as 116 basic kernels. In MKL, a liner combination of 116 basic kernels considered the final synthetic nuclear space.

A leave-one-out cross-validation was applied across participants to obtain estimates for each participant. The 89 participants were divided into 89 groups of 88 participants. Once trained, the simple MKL inform a prediction for the cognition scores of the 88 participants based on their neuroimaging data (eg. mALFF map). Accuracy of simple MKL prediction was calculated, defined as the Pearson’s correlation coefficient and mean squared error (MSE) between actual and predicted values of the cognitive scores.

In simple MKL regression analysis, there are five steps to analysis including data and design, prepare feature set, specify model, run model, display result.

Frist, in “data and design” step, we use 89 resting -state whole-brain mALFF data, and we define the SA score as regression targets, and we also add sex, age, education level, head motion and CAPS scores as covariates. Then we add whole-brain AAL mask into the model.

Then, in “prepare feature set” step, put all the data from the first step and the not detrend and not scaling the mALFF data, and build one kernel per AAL region in this step. There is one modality to concatenate/combine in this step, and after all the parameters is done, the model come to build kernel and data matrix.

Next, in “specify model "step, we put the model we get from step 2, and we defined the feature of regression in this step. We use Kernel Ridge Regression to calculate the correlation of the SA score and mALFF data from 89 individuals. And then we use leave one out way to do the cross-validation scheme, and we use mean centre features using analysis and to realize the data operations. We also regressed out the HD covariate in this step. After that, we applied the step of specify model.

In the “Run model” step, we analysis the model in PRT we set above.

In the “Display result” step, we use the model run out and do the 1000 permutation test. After that, we compute the weights of each ROI in the prediction model by the Pronto.

In the SVM classify analysis, there are five steps to analysis including data and design, prepare feature set, specify model, run model, display result.

Frist, in data and design step, we use 78 resting -state whole-brain mALFF data in two groups, and we also add sex, age, education level, and head motion as covariates. Then we add whole-brain AAL mask into the model.

Then, in “prepare feature set” step, put all the data from the first step and the not detrend and not scaling the mALFF data, and build one kernel per AAL region in this step. There is one modalities to concatenate/combine in this step, and after all the parameters is done, the model come to build kernel and data matrix.

Next, in “specify model” step, we put the model we get from step 2, and we defined the feature of the classification model in this step. We use Binary support vector machine to realize the classification. And then we use leave one out way to do the cross-validation scheme, and we use mean centre features using to realize the data operations. We also regressed out the HD covariate in this step. After that, we applied the step of specify model.

In the “Run model” step, we analysis the model in PRT we set above.

In the “Display result” step, we use the model run out and do the 1000 permutation test.

1. Univariate SPM analysis

We used multiple regression model in SPM8 software to identify voxel in mALFF maps which showed a significant association with memory subscale scores that is significantly correlated with CAPS scores. We corrected for multiple comparisons using Family Wise Error (FEW). Statistical inferences were made at P<0.05.

8. Weighted sorting and expected sorting table

**Table 1** Weighted sorting and expected sorting table

**(**Neuroanatomical Regions With a contribution to frame less than 2% across All Regions for the Resting-state Functional MRI-based MKL Used to Accurately Predict SA)

| AAL | Brain region | Contribution proportion (%) | Number  of voxels (vox) | Desired ordering |
| --- | --- | --- | --- | --- |
| 93 | Cerebelum_Crus2_L | 1.492582 | 518 | 15.595506 |
| 92 | Cerebelum_Crus1_R | 1.015594 | 596 | 16.808989 |
| 45 | Cuneus_L | 1.003709 | 460 | 17.044944 |
| 69 | Paracentral_Lobule_L | 0.967051 | 309 | 18.741573 |
| 51 | Occipital_Mid_L | 0.865177 | 950 | 23.05618 |
| 57 | Postcentral_L | 0.828141 | 1100 | 21.292135 |
| 50 | Occipital_Sup_R | 0.252205 | 391 | 31.876404 |
| 91 | Cerebelum_Crus1_L | 0.117359 | 665 | 78.213483 |
| 86 | Temporal_Mid_R | 0.025849 | 1125 | 86.258427 |
| 19 | Supp_Motor_Area_L | 0.01901 | 615 | 31.617978 |

Cuneus_L ,Left cuneus; Paracentral_Lobule_L ,Left paracentral lobule; Occipital_Mid_L ,Left middle occipital gyrus; Postcentral_L ,Left postcentral gyrus; Occipital_Sup_R ,Right superior occipital gyrus; Temporal_Mid_R ,Right middle temporal gyrus; Supp_Motor_Area_L ,Left supplementary motor area

9. The correlation between CAPS score and cognitive function

| Value | CAPS | | | |
| --- | --- | --- | --- | --- |
|  | Correlation | | | *p* |
| Overall Full-Scale Memory quotient | | -0.15 | 0.19 | |
| Auditory memory index | | -0.02 | 0.85 | |
| Visual memory index | | -0.20 | 0.07 | |
| Visual work memory index^b^ | | -0.22 | 0.05 | |
| Instant memory index ^b^ | | -0.17 | 0.12 | |
| Delayed memory index^b^ | | -0.06 | 0.58 | |

10. Frequency distribution of SA scores


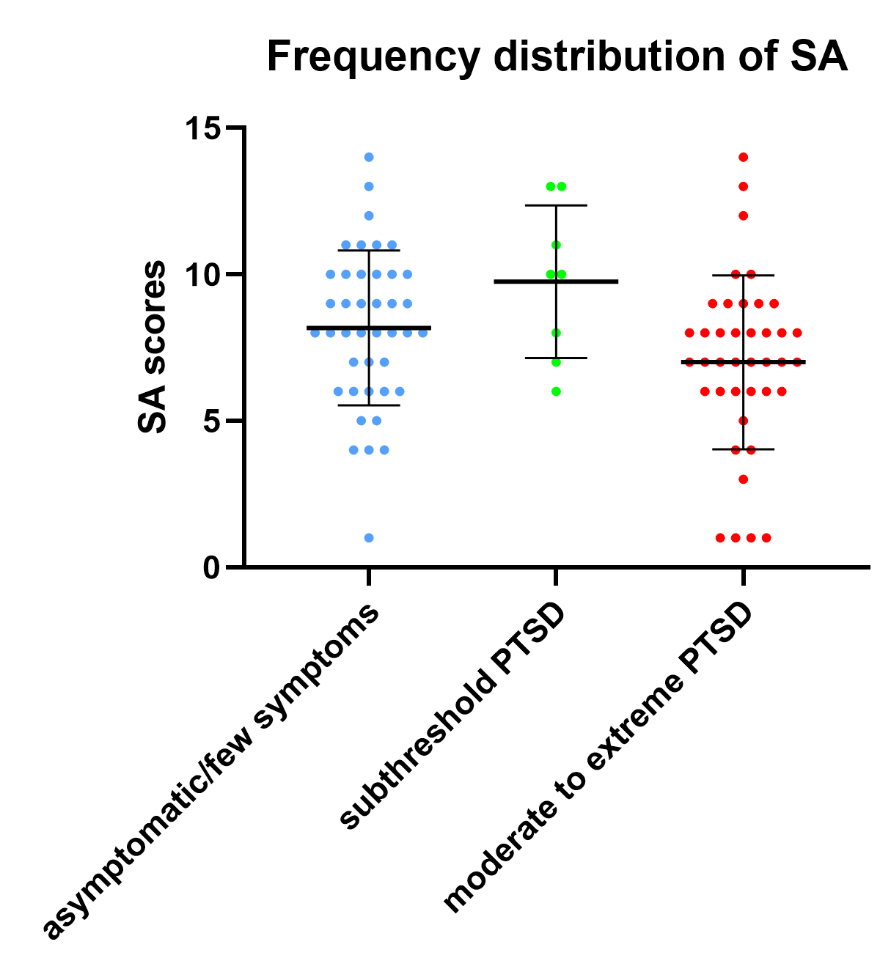


**PTSD**, post-traumatic stress disorder; **CAPS**, Clinician-Administered Posttraumatic Stress Disorder Scale

11. Simple MKL Analysis of whole brain Rs-mALFF predicting the CAPS scores

The application of MKL to the whole-brain Rs-mALFF data did not allowed quantitative prediction of CAPS scores, for it don’t have statistically significant accuracy (correlation=0.06, P-value=0.27; mean squared error=1468.62, P-value=0.64).

12. Exclude the individuals with SA=1

We speculated that individuals with very low SA scores might influence the predictive function of the frame, thus, we excluded individuals with SA in one score, and the effect size and the P value of the frame is much better (correlation=0.36, P-value=0.01; mean squared error=5.09, P-value=0.01) (corrected for multiple comparisons using the permutation test, both P-value <0.05) (figure 2). Because SA=1 is an extremely low score that means individuals answered totally wrong. Although our assessment is applied one-to-one, and we also observed whether they were carefully completed as quality control, and we also give examples before the assessment to make sure the subjects understand the assessment, they may also misunderstand. And they take bus from Qingchuan to Chengdu, which cost them 5 hours. And as the study using WSM-IV an only be done on Saturdays or Sundays when there are no clinical patients, thus, some of the individuals maybe tired which influence their SA score. However, we don’t have any evidence to exclude them because of the low SA score, it indicated that sometimes it is hard to get the accurate cognitive condition, so that estimate SA score by mALFF is important in clinical practice. And we also need to increase the sample size of the current study then the outliner can have less influence.


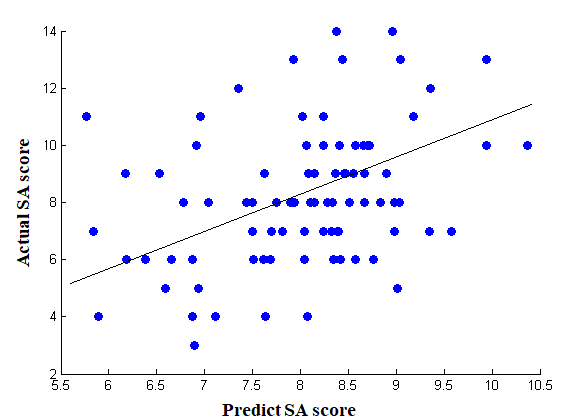


13. Confirming the effect of exclusion criteria

To confirm the effect of exclusion criteria, we reanalyzed the Data Preprocessing step, data from 4 trauma survivors were discarded when the mean framewise displacement (FD) exceeded 0.25mm or when translational or rotational parameters exceeded ± 3.0 mm or ± 3.0°. As scrubbing is not recommended for mALFF^[4]^, we regress out the mean framewise displacement (FD) in simple MKL regression analysis. We found the results were robust after reanalysis. The application of simple MKL to the whole-brain Rs-mALFF data allowed quantitative prediction of SA scores with statistically significant accuracy (correlation=0.24, P-value=0.03; mean squared error=8.41, P-value=0.04) (corrected for multiple comparisons using the permutation test, both P-value <0.05).


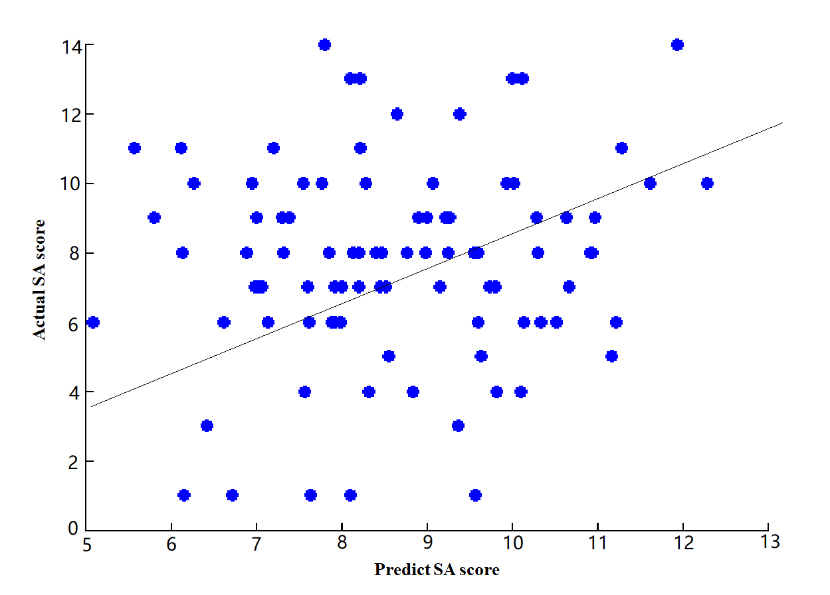


**Reference**

[1] Alain Rakotomamonjy FRB, Canu S. SimpleMKL. Journal of Machine Learning Research. 9,2008. 2491-2521.

[2] Lanckriet, T. De Bie, N. Cristianini, M. Jordan, and W. Noble. A statistical framework for genomic data fusion. Bioinformatics, 20:2626–2635, 2004a .

[3] Lanckriet GRG, Christianini N, Bartlett PL, Ghaoui LE, Jordan MI. Learning the Kernel Matrix with Semi-Definite Programming. 2002 : 323-330.

[4] Yan CG, Cheung B, Kelly C, et al. A comprehensive assessment of regional variation in the impact of head micromovements on functional connectomics. Neuroimage, 2013,76:183-201.
